# Inhibition of mTOR prevents glucotoxicity-mediated increase of SA-beta-gal, p16^INK4a^, and insulin hypersecretion, without restoring electrical features of mouse pancreatic islets

**DOI:** 10.1007/s10522-024-10107-9

**Published:** 2024-05-15

**Authors:** Tereso J. Guzmán, Nina Klöpper, Carmen M. Gurrola-Díaz, Martina Düfer

**Affiliations:** 1 Department of Pharmacology, Institute of Pharmaceutical and Medicinal Chemistry, University of Münster, Corrensstraße 48, 48149 Münster, Germany; 2 Departamento de Biología Molecular y Genómica, Universidad de Guadalajara, Instituto de Investigación en Enfermedades Crónico-Degenerativas, Centro Universitario de Ciencias de la Salud, 44340 Guadalajara, Jalisco México

**Keywords:** Hyperglycemia, Oxidative stress, Sirolimus, Pancreatic beta-cells, MIN6 cells, Microelectrode arrays, Senescence, Glucose-stimulated insulin secretion

## Abstract

An over-activation of the mechanistic target of rapamycin (mTOR) pathway promotes senescence and age-related diseases like type 2 diabetes. Besides, the regenerative potential of pancreatic islets deteriorates with aging. Nevertheless, the role of mTOR on senescence promoted by metabolic stress in islet cells as well as its relevance for electrophysiological aspects is not yet known. Here, we investigated whether parameters suggested to be indicative for senescence are induced in vitro in mouse islet cells by glucotoxicity and if mTOR inhibition plays a protective role against this. Islet cells exhibit a significant increase (~ 76%) in senescence-associated beta-galactosidase (SA-beta-gal) activity after exposure to glucotoxicity for 72 h. Glucotoxicity does not markedly influence p16^INK4a^ protein within 72 h, but p16^INK4a^ levels increase significantly after a 7-days incubation period. mTOR inhibition with a low rapamycin concentration (1 nM) entirely prevents the glucotoxicity-mediated increase of SA-beta-gal and p16^INK4a^. At the functional level, reactive oxygen species, calcium homeostasis, and electrical activity are disturbed by glucotoxicity, and rapamycin fails to prevent this. In contrast, rapamycin significantly attenuates the insulin hypersecretion promoted by glucotoxicity by modifying the mRNA levels of *Vamp2* and *Snap25* genes, related to insulin exocytosis. Our data indicate an influence of glucotoxicity on pancreatic islet-cell senescence and a reduction of the senescence markers by mTOR inhibition, which is relevant to preserve the regenerative potential of the islets. Decreasing the influence of mTOR on islet cells exposed to glucotoxicity attenuates insulin hypersecretion, but is not sufficient to prevent electrophysiological disturbances, indicating the involvement of mTOR-independent mechanisms.

**Supplementary Information:**

The online version contains supplementary material available at 10.1007/s10522-024-10107-9.

## Introduction

During the aging process, the accumulation of oxidative damage leads to cellular senescence and contributes to the development of age-related diseases (Childs et al. 2015; Kaur and Farr 2020). Accordingly, the risk to develop type 2 diabetes (T2D) increases with age (Magliano and Boyko 2021). Hormone secretion by pancreatic islet cells plays a pivotal role in regulating and maintaining normal blood glucose levels. Particularly, as age increases, disturbances of the mass and/or function of pancreatic beta-cells combined with cellular failure caused by over-nutrition, have strong implications on T2D pathophysiology.

An over-activation of the mechanistic target of rapamycin (mTOR) signaling pathway is suggested to be involved in the promotion and progression of senescence and organismal aging (Blagosklonny 2022; Guillen and Benito 2018). In line with this, rapamycin (syn. sirolimus), a well-known inhibitor of the mTOR complex, has been shown to induce autophagy, increase longevity, and exert beneficial metabolic effects in several in vitro and in vivo studies (Bitto et al. 2016; Chang et al. 2009; Schreiber et al. 2015; Yang et al. 2012). Likewise, diverse pharmacological inducers of autophagy, which may counteract and decrease the hallmarks of senescence and aging, are proposed and tested as potential therapies to extend not only the lifespan, but the healthspan as well (Hansen et al. 2018; Nakamura and Yoshimori 2018). The study and potential therapeutic use of these compounds is highly relevant due to prospective reductions in the economic burden caused by diseases occurring in the elderly population.

In the case of pancreatic islet cells, previous studies indicate an age-dependent decline in the regenerative capacity of beta-cells from humans and rodents. This has been correlated with an augmented expression of senescence markers such as the senescence-associated beta-galactosidase (SA-beta-gal) and the p16^INK4a^ protein (Gregg et al. 2012; Sone and Kagawa 2005; Tschen et al. 2009). Moreover, the involvement of pancreatic beta-cell senescence in the pathology of diabetes has been recently addressed, and there is evidence that senescence represents a novel and relevant target for diabetes management (Aguayo-Mazzucato et al. 2019; Thompson et al. 2019). Notwithstanding, despite data showing that beta-cell senescence plays an active role in dysglycemia and diabetes development, it has also been proposed that either aging of islets may not be critical for diabetes pathogenesis or that senescence may turn out beneficial for beta-cells function (Almaca et al. 2014; Helman et al. 2016; Kehm et al. 2018). In summary, the significance of beta-cell senescence for altered blood glucose homeostasis requires to be further investigated.

Regarding the importance of mTOR signaling, these pathways have mostly been studied under physiological conditions, but little is known about the influence of mTOR inhibition under metabolic stress-induced senescence in pancreatic beta-cells. Besides, the influence of manipulating the mTOR pathway on the electrophysiological features of islet cells exposed to a senescence-promoting milieu has not been studied. Here, we investigated whether senescence is induced in mouse islet cells exposed in vitro to a metabolic stress-promoting scenario and whether inhibition of mTOR-regulated signaling pathways exerts a protective role against these experimental conditions. To address this, we focused on evaluating the SA-beta-gal activity and the p16^INK4a^ protein immunostaining in mouse islet cells cultured under glucotoxic conditions in the presence or absence of rapamycin. To gain insight into the functional consequences of senescence for pancreatic islets and to identify if rapamycin treatment exhibits any protective effects, we evaluated reactive oxygen species (ROS) production, intracellular calcium levels, electrical activity, and insulin secretion of cells and whole islets exposed to the pro-senescence experimental conditions.

## Methods

### Chemicals

Salts, glucose, bovine serum albumin, dimethyl sulfoxide (DMSO), and 2’, 7’-dichlorodihydrofluorescein diacetate (DCDHF-DA) were obtained from Sigma-Aldrich (Taufkirchen, Germany) or Diagonal (Münster, Germany). DMEM and RPMI 1640 medium, fetal bovine serum, and antibiotics were from Life Technologies (Darmstadt, Germany). Rapamycin was obtained from Enzo Life Sciences (Lausen, Switzerland), and 5-dodecanoylaminofluorescein di-beta-D-galactopyranoside (C_12_FDG), rabbit anti-mouse p16^INK4a^ (catalogue: ab211542) and goat anti-rabbit IgG H&L (Alexa Fluor® 488, catalogue: ab150077) were from Abcam (Cambridge, United Kingdom). MitoSOX was obtained from Molecular Probes (Eugene, USA), and fura-2-acetoxymethylester (Fura-2 AM) from Biotrend (Köln, Germany).

### Cell culture

For experiments with primary cells, we isolated pancreatic islets from mice. Briefly, pancreatic tissue from adult C57BL/6N male and female mice (Charles River, Sulzfeld, Germany) was digested by collagenase P (Roche Diagnostics, Mannheim, Germany). Dispersed islet cells were obtained by digestion with trypsin (Sigma-Aldrich, Taufkirchen, Germany). Islets and dispersed islet cells were cultured overnight in RPMI 1640 medium (11.1 mM glucose) supplemented with 10% fetal bovine serum, 100 U/ml penicillin, and 100 µg/ml streptomycin before the experiments were performed. The care and use of laboratory animals were adhered to the German laws (Az.: 53.5.32.7.1/MS-12668, Health and Veterinary Office Münster, Germany).

For maintenance and expansion of the insulin-producing MIN6 cell line, we cultured the cells in DMEM (25 mM glucose) supplemented with 15% heat-inactivated, fetal bovine serum, 100 U/ml penicillin, and 100 µg/ml streptomycin. Culture medium was exchanged every 3rd to 4th day and cells were split when a 70 − 80% confluence was reached. Before performing experiments, we seeded and incubated the cells overnight in standard culture medium. All cell cultures were maintained in a humidified atmosphere at 37 °C and 5% CO_2_.

### Experimental induction of senescence and pharmacological treatments

Islets or dispersed islet cells were exposed to 33 mM glucose for 72 h or 7 days. Alternatively, the cells were exposed to sub-lethal hydrogen peroxide concentrations (1, 10 or 30 µM) for 2 h and allowed to recover for 72 h. Unless indicated otherwise, standard culture medium containing 10 mM glucose was used as control, which is known to keep the apoptosis rates of murine islets and islet cells at a low level (Ramirez-Dominguez 2016).

In experiments performed with MIN6 cells, cells were exposed to 33 mM glucose for 72 h and compared to cells cultured in medium supplemented with 5 mM glucose as a control condition.

Rapamycin was added to the respective culture medium at the indicated times and concentrations to inhibit mTOR signaling. Rapamycin was no longer present in the respective buffer, bath or staining solutions. For control conditions, cells were treated with an equal volume of the vehicle (0.1% v/v DMSO).

### Evaluation of senescence-associated beta-galactosidase (SA-beta-gal)

To analyze the activity of SA-beta-gal in primary islet or MIN6 cells, the SA-beta-gal fluorogenic substrate C_12_FDG was employed as reported previously (Debacq-Chainiaux et al. 2009; Wang et al. 2013). Cells were cultivated on glass coverslips. After the treatment periods, cells were stained with 33 µM C_12_FDG diluted in fresh medium and incubated at 37 °C in a humidified atmosphere and 5% CO_2_ for 4 h. The cells were washed three times with phosphate buffered saline (PBS) and the images were acquired using a 40 × oil objective in an inverted fluorescence microscope (Nikon Eclipse Ti, Nikon Instruments Inc., Melville, USA) coupled with a CoolSNAP HQ2 digital camera (Teledyne Photometrics, Tucson, USA). We captured ten different fields per coverslip from each of the duplicates included per condition in every experiment. Cell fluorescence was quantified by the NIS elements software (Nikon Instruments Inc., Melville, USA). To perform an objective comparison and minimize the influence of the variability among the basal raw fluorescence in different experiments (λ_exc_: 480 nm, λ_em_: 535/30 nm; Online Resource, Figure S1), we analyzed the mean fluorescence values as percentage of the control condition of each experiment.

### Expression and cellular localization of the p16^INK4a^ senescence protein

For immunostaining of the p16^INK4a^ senescence protein, islet cells were washed three times with PBS and fixed by 3% paraformaldehyde at room temperature (RT) for 1 h after the respective treatments. Thereafter, the samples were washed with PBS, incubated with 100 mM glycine (RT, 10 min), washed again, blocked with goat serum (10%, v/v in PBS, RT, 10 min) and cells were permeabilized with Triton X-100 (0.25%, v/v in PBS, 10 min, RT). A rabbit anti-p16 primary antibody (1:50 in 10% goat serum) was applied for 2 h at RT. After washing with PBS, the samples were incubated with a goat anti-rabbit IgG secondary antibody (1:500, 1 h, RT). Finally, cells were washed with PBS and Fluoroshield with DAPI mounting medium was added (Sigma-Aldrich, Taufkirchen, Germany). Confocal images were acquired by a 60 × oil objective and an iMic confocal microscope (FEI, Munich, Germany) coupled with an ORCA-Flash 4.0 camera (Hamamatsu Photonics, Hamamatsu, Japan, λ_exc_: 480 nm). For evaluation of p16^INK4a^, we quantified the cytosolic and nuclear integrated density of the fluorescence signal using the ImageJ software (National Institutes of Health, Bethesda, USA). Nuclear localization was defined by DAPI co-staining and the cytosolic fluorescence was calculated by subtracting the nuclear area from whole-cell fluorescence. Negative staining controls, with cells incubated under the same experimental conditions, were stained in the absence of the primary antibody and analyzed similarly for correction of fluorescence values (Online Resource, Figure S2).

### Oxidative status, mitochondrial superoxide levels, and ROS accumulation kinetics

DCDHF-DA was used for evaluation of the general oxidative state of the cells and the ROS accumulation kinetics. Briefly, 50,000 MIN6 cells/well were seeded in a tissue-culture treated, 96-well black plate, and cultivated at the indicated conditions. Cells were washed with PBS and stained by 20 μM DCDHF-DA in a Krebs–Ringer-HEPES (KRH) buffer containing 122 mM NaCl, 4.7 mM KCl, 1.1 mM MgCl_2_, 2.5 mM CaCl_2_, 10 mM HEPES, 0.5% (v/v) BSA, and 3 mM glucose for 30 min at 37 °C. After replacing the staining solution by fresh KRH buffer, the basal oxidative status was determined by a fluorometer (BMG CLARIOstar, BMG LabTech GmbH, Ortenberg, Germany, λ_exc_: 483/14 nm). To assess ROS production kinetics, we increased the glucose concentration from 3 to 15 mM and measured the fluorescence intensity every 5 min during a 60-min period. The area under the curve (AUC) was calculated using the trapezoidal rule formula.

For the evaluation of mitochondrial superoxide levels, MIN6 cells were treated as described above but loaded with 5 µM MitoSox for 20 min. Fluorescence intensity values (λ_exc_: 510/15 nm) were normalized to the control condition.

### Intracellular Ca^2+^ levels and oscillatory pattern assessment

Whole mouse islets were put on poly-L-lysine-coated glass coverslips and cultured under the indicated conditions for 72 h. Thereafter, the islets were loaded with 5 μM Fura-2 AM in a bath solution containing 140 mM NaCl, 5 mM KCl, 1.2 mM MgCl_2_, 2.5 mM CaCl_2_, 10 mM HEPES, and 10 mM glucose for 30 min at 37 °C. Afterwards, the islets were perifused with the bath solution (10 mM glucose) for 20 min. At the end of each experiment glucose concentration was lowered to 3 mM.

Fluorescence induced by alternating excitation wavelengths of 340 and 380 nm (F_340_/F_380_; VisiChrome High Speed Polychromator, Visitron Systems, Puchheim, Germany) was monitored by a fluorescence microscope (Nikon Eclipse Ti2, Nikon Instruments Inc., Melville, USA) coupled to a QImaging Retiga R1 digital camera (Teledyne Photometrics, Tucson, USA). The AUC of cytosolic Ca^2+^ for the stimulatory (10 mM) or the non-stimulatory (3 mM) glucose concentration was calculated for a period of 15 and 5 min, respectively.

For evaluation of Ca^2+^ levels in a hyperpolarized state, 250 μM diazoxide was added during the loading period with Fura-2 AM (30 min, 37 °C). The cytosolic Ca^2+^ was evaluated for a period of 2 min in the continued presence of diazoxide and 10 mM glucose and the average of the F_340_/F_380_ ratio was calculated.

### Electrical activity recordings

The electrical activity of pancreatic islets was monitored by the microelectrode array system MEA2100 (MultiChannel Systems, Reutlingen, Germany). After preparation, the islets were allowed to recover in standard culture medium for 48 h. Next, the islets were put on MEAs coated with Matrigel, allowed to attach for 24 h, and thereafter cultivated under the indicated experimental conditions. After the respective treatments the electrical activity evoked by bath solution (see above) with 10 mM glucose was recorded for 30 min followed by a reduction of glucose to 3 mM glucose for 10 min. For data analysis, we calculated the fraction of plateau phase (FOPP), i.e., the fraction of time as percentage that exhibits electrical bursts. Data were acquired at sampling frequency of 1000 Hz with the Beta-Screen Experimenter v.2.12.0 software, low-pass filtered at 20 Hz, and analyzed with the Beta-Screen Analyzer v.2.15.2 software (MultiChannel Systems, Reutlingen, Germany).

### Ex vivo glucose-stimulated insulin secretion

For the assessment of the glucose-stimulated insulin secretion and insulin content, islets were exposed to glucotoxicity in the presence or absence of 1 nM rapamycin for 7 days. As a reference islets were cultured overnight in standard culture medium. After silencing the insulin secretion with KRH buffer (0.5% BSA v/v, composition see above) containing 5.6 mM glucose for 1 h and 3 mM glucose for 30 min at RT, the islets (5 per condition, in duplicate) were incubated with 15 mM or 3 mM glucose (KRH, 0.5% BSA v/v) for 1 h at 37 °C. The supernatant was collected, the islets were lysed with acidic ethanol, and samples were stored at − 20 °C until analysis. Insulin was determined by ELISA (Mercodia, Uppsala, Sweden) following the manufacturer’s instructions.

### RNA isolation, cDNA synthesis, and real-time PCR

After the respective treatments total RNA of islets was isolated with the Nucleospin RNA XS kit (Macherey–Nagel, Düren, Germany) following the instructions provided by the manufacturer. cDNA was synthesized of 200 ng of total RNA with the iScript cDNA synthesis kit (Bio-Rad, Hercules, CA). Real-time PCR assays were performed with iQ Sybr Green supermix (Bio-Rad, Hercules, CA) in a LightCycler 96 system (Roche, Mannheim, Germany). Amplification reactions were carried out in triplicate and negative controls were included in every assay by replacing cDNA by molecular grade water. Relative gene expression levels were analyzed by the 2^−ΔΔCt^ method using *Actb* as reference. The nucleotide sequences of the primers are shown in Table S1 (Online Resource). The primers were ordered from Microsynth (Balgach, Switzerland).

### Statistical analysis

After evaluating data distribution by the Shapiro–Wilk test, inferential statistical analyses were performed with parametric or non-parametric tests, accordingly. Data are presented as the mean ± standard error of the mean (SEM), unless indicated otherwise, from experiments of at least three independent mouse preparations or passages of MIN6 cells. Experimental datasets were compared by one-way analysis of variance (ANOVA) or Kruskal–Wallis test followed by Newman-Keuls’ or Dunn’s post hoc test, respectively. Statistical significance was considered when *p* values were < 0.05.

## Results

### Exposure to glucotoxicity, but not to hydrogen peroxide, increases SA-beta-gal activity of islet cells and insulin-producing MIN6 cells in an mTOR-dependent manner

**Fig. 1:**
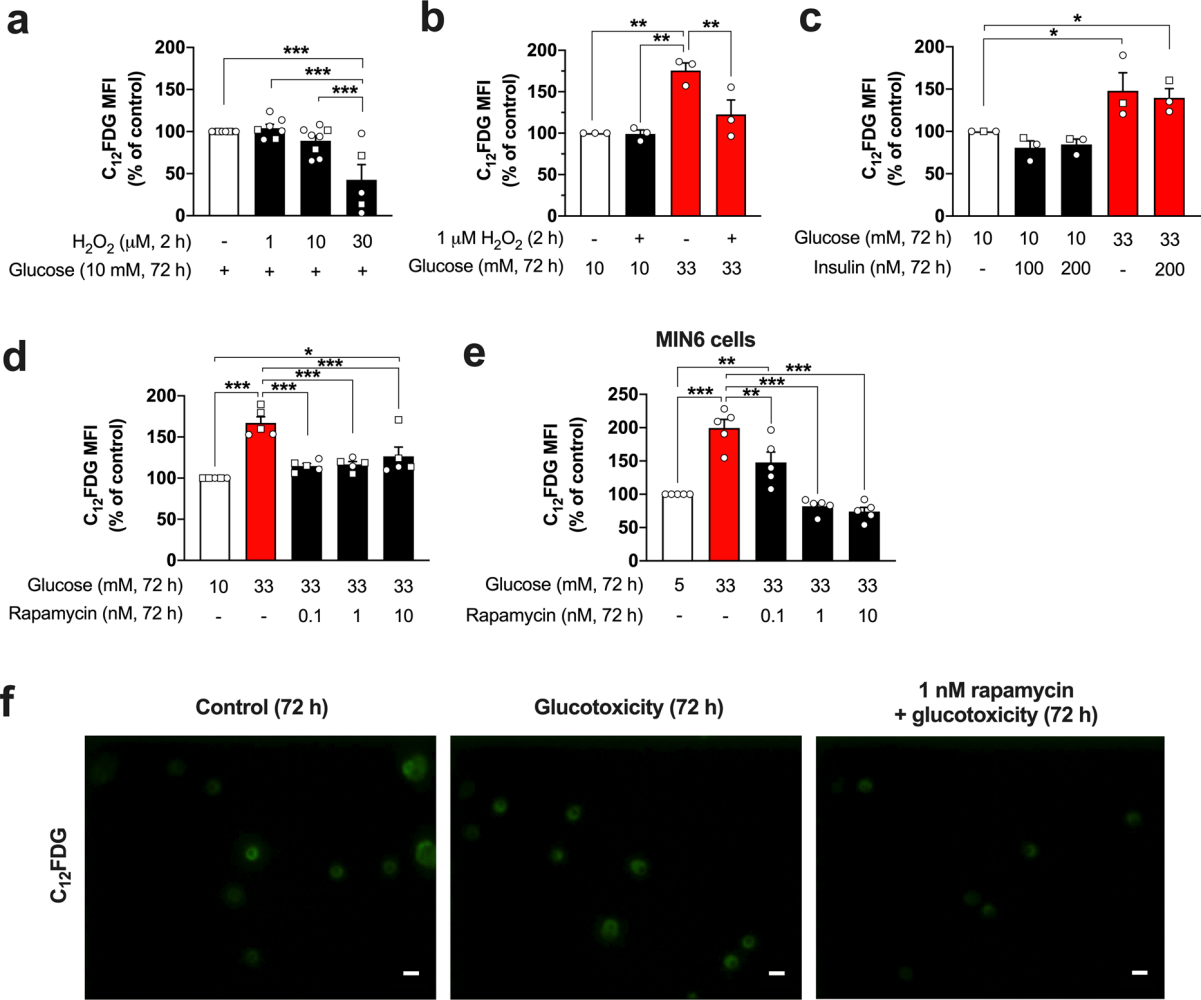
Influence of glucotoxicity and mTOR inhibition on the SA-beta-gal activity of primary islet cells. After a 2 h-exposure to hydrogen peroxide, the mouse islet cells were cultured for additional 72 h in standard culture medium (**a**). Alternatively, after the exposure to hydrogen peroxide (1 µM, 2 h) or vehicle, the islet cells were cultured for 72 h in a standard or glucotoxic (33 mM glucose) condition (**b**). Hydrogen peroxide does not increase SA-beta-gal but even reduces it at a concentration of 30 µM (**a**). In contrast, 33 mM glucose induces a significant rise in the activity of this senescence marker (**b**). The effects of insulin on SA-beta-gal were evaluated in standard or glucotoxic conditions, without showing any effect (**c**). Inhibition of mTOR signaling by rapamycin (0.1, 1 and 10 nM, 72 h) prevents the effects of glucotoxicity in islet cells (**d**) and MIN6 cells (**e**). Representative images of SA-beta-gal staining by C_12_FDG of islet cells cultured for 72 h under control, glucotoxic, or glucotoxic condition in the presence of rapamycin (**f**). MFI: mean fluorescence intensity. The number of independent mouse preparations (~ 7 months of age, **a − d**) or experiments (**e**) is indicated by the symbols. In mouse preparations, circles and squares represent female and male mice, respectively. *p < 0.05; **p < 0.01; ***p < 0.001. Scale bar (**f**) = 10 µm

As an initial attempt to promote the induction of senescence in primary mouse islet cells, hydrogen peroxide was used as a direct source of oxidative stress. Mouse pancreatic islet cells were exposed to hydrogen peroxide concentrations of 1, 10 and 30 µM for 2 h followed by a recovery period of 72 h in standard culture conditions. Contrary to our expectations, the exposure of islet cells to this oxidant had either no influence or even a concentration-dependent decreasing effect on the SA-beta-gal activity determined as C_12_FDG fluorescence (Fig. 1a).

Since hydrogen peroxide was not suitable for promoting the increase in the activity of the marker SA-beta-gal, a different experimental approach was used in the form of a nutrient excess-driven metabolic stress condition. For these assays, the islets cells were cultured in medium with a high-glucose concentration of 33 mM glucose for 72 h. Remarkably, the exposure of mouse islet cells to this glucotoxic condition leads to a significant increase in the level of SA-beta-gal activity compared to control cells (~ 76%, *p* < 0.01). To clarify the overall influence of hydrogen peroxide, we evaluated the effects of combining both scenarios, the oxidant (1 µM hydrogen peroxide) and the glucotoxicity (33 mM glucose). Interestingly, the 2-h pre-treatment with hydrogen peroxide does not affect SA-beta-gal activity but significantly attenuates the increase in SA-beta-gal activity that is induced by the following 72 h-culture period with medium supplemented with 33 mM glucose (Fig. 1b).

Considering that the physiological role of beta-cells relies on their capacity to secrete insulin in response to a glucose stimulus, an autocrine effect of insulin might be involved in the regulation of SA-beta-gal. Therefore, we investigated whether the treatment with exogenous insulin (100 or 200 nM) had an influence on the induction of SA-beta-gal activity in islet cells exposed to glucotoxicity. Exogenous insulin treatment (72 h, standard medium or medium containing 33 mM glucose) does not alter the effects of glucotoxicity on the SA-beta-gal induction or influence SA-beta-gal per se (Fig. 1c).

The inhibition of mTOR has been suggested to exert a protective role against senescence, and recent studies have shown beneficial effects on metabolism, lifespan, and healthspan (Hansen et al. 2018; Nakamura and Yoshimori 2018). Interestingly, we found that the concomitant treatment of islet cells with rapamycin (0.1, 1, and 10 nM) and glucotoxicity prevents the increase of SA-beta-gal activity (Fig. 1d, f). To corroborate our data, we performed analogous experiments with insulin-producing MIN6 cells. Similar to primary islet cells, exposing the MIN6 cells to glucotoxicity leads to a significant increase of SA-beta-gal activity and inhibiting mTOR signaling by rapamycin protects against this effect in a concentration-dependent manner (Fig. 1e). Noteworthy, on both primary and MIN6 cells, a rapamycin concentration in the picomolar range is sufficient to protect against the glucotoxicity-induced SA-beta-gal activation.

### The p16^INK4a^ senescence protein expression depends on mTOR signaling and is significantly increased by prolonged exposure to glucotoxicity

**Fig. 2:**
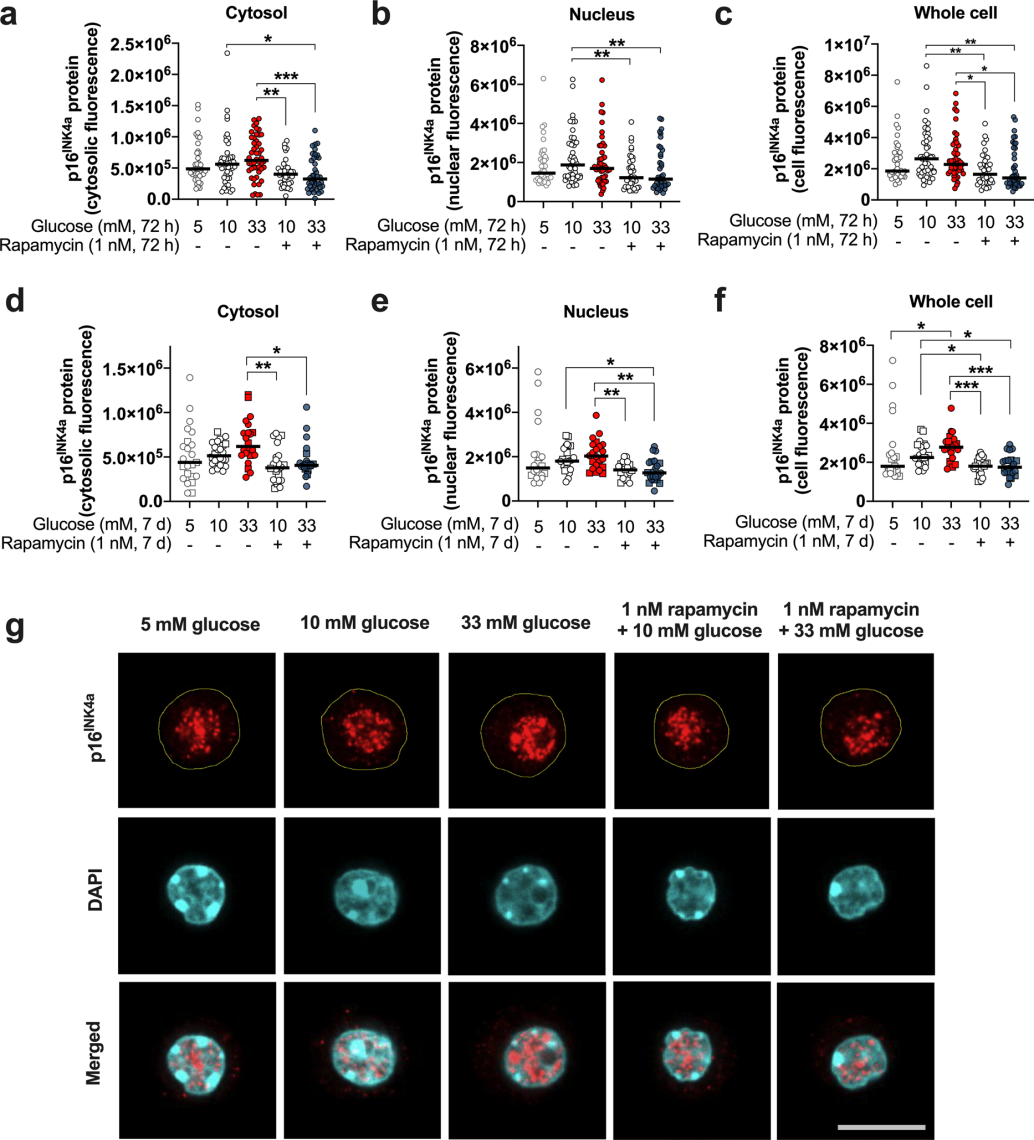
The level of the p16^INK4a^ protein immunostaining in mouse islet cells depends on glucose and mTOR activation. The p16^INK4a^ protein was detected in the cytosol (**a**, **d**), nucleus (**b**, **e**), and whole cell (**c**, **f**) in pancreatic mouse islets cells exposed to glucotoxicity in the presence or absence of 1 nM rapamycin for 72 h (**a**−**c**) or 7 days (**d**−**f**). The p16^INK4a^ immunostaining displays a glucose-dependent trend to increase in cytosol and nucleus that is more prominent and even significant for the whole cell level after a 7 days-exposure to glucotoxicity. Rapamycin reduces p16^INK4a^ immunostaining irrespective of time and glucose concentration. Representative images of the p16^INK4a^ cellular localization and immunofluorescence in islet cells exposed to glucotoxicity in the presence or absence of rapamycin for 7 days (**g**, border of the cells highlighted in yellow in the upper traces). **a−c**: Circles indicate cells evaluated from three independent female mouse preparations; **d**−**f**: the circles and squares represent individual cells analyzed from three independent mouse preparations, two females and one male, respectively (~ 8-months of age). *p < 0.05; **p < 0.01; ***p < 0.001. Scale bar (**g**) = 10 µm

Considering that glucotoxicity was able to promote an increase of SA-beta-gal levels in the islet cells, we decided to evaluate the influence of this deleterious condition on the expression and localization of the p16^INK4a^ protein, a senescence marker. Remarkably, our data show that the p16^INK4a^ protein immunostaining exhibits heterogenous levels in adult islet cells cultured in standard medium supplemented with 5 or 10 mM glucose. Moreover, the immunofluorescence staining of the protein is found mainly in the nuclear compartment (Fig. 2b), and the nucleus exhibited two- to four-times the p16^INK4a^ protein compared to the cytosol (Fig. 2a). Contrary to our expectations, the 72 h-exposure of the cells to glucotoxicity does not significantly increase the levels of p16^INK4a^ immunostaining irrespective of the cellular compartment, although a slight trend towards increase was found at 10 and 33 mM glucose compared to 5 mM glucose (Fig. 2a−c). Rapamycin treatment, on the other hand, significantly reduces the p16^INK4a^ immunostaining in the cytosolic and nuclear compartment and maintains the fluorescence levels similar to those observed after culture in medium supplemented with 5 mM glucose (*p* < 0.01, Fig. 2a−c).

Since the expression of the p16^INK4a^ senescence protein was not markedly influenced by glucotoxicity after 72 h, we prolonged the exposure to this condition to 7 days. Noteworthy, the whole cell p16^INK4a^ content is significantly increased after application of 33 mM glucose for 7 days compared to cells cultivated in medium with 5 mM glucose and the signal is less heterogeneous and more consistent among the cells (Fig. 2d−f). Similar to the shorter incubation period, mTOR inhibition significantly lowers p16^INK4a^ immunostaining in the whole cell, the nucleus, and the cytosol (Fig. 2d−f). No difference in the cellular distribution of p16^INK4a^ is observed under the different experimental conditions evaluated as nucleus-to-cytosol ratios (analysis not shown).

### Inhibition of mTOR partly prevents the effect of glucotoxicity on the oxidative state of MIN6 cells without preserving mitochondrial superoxide production

**Fig. 3:**
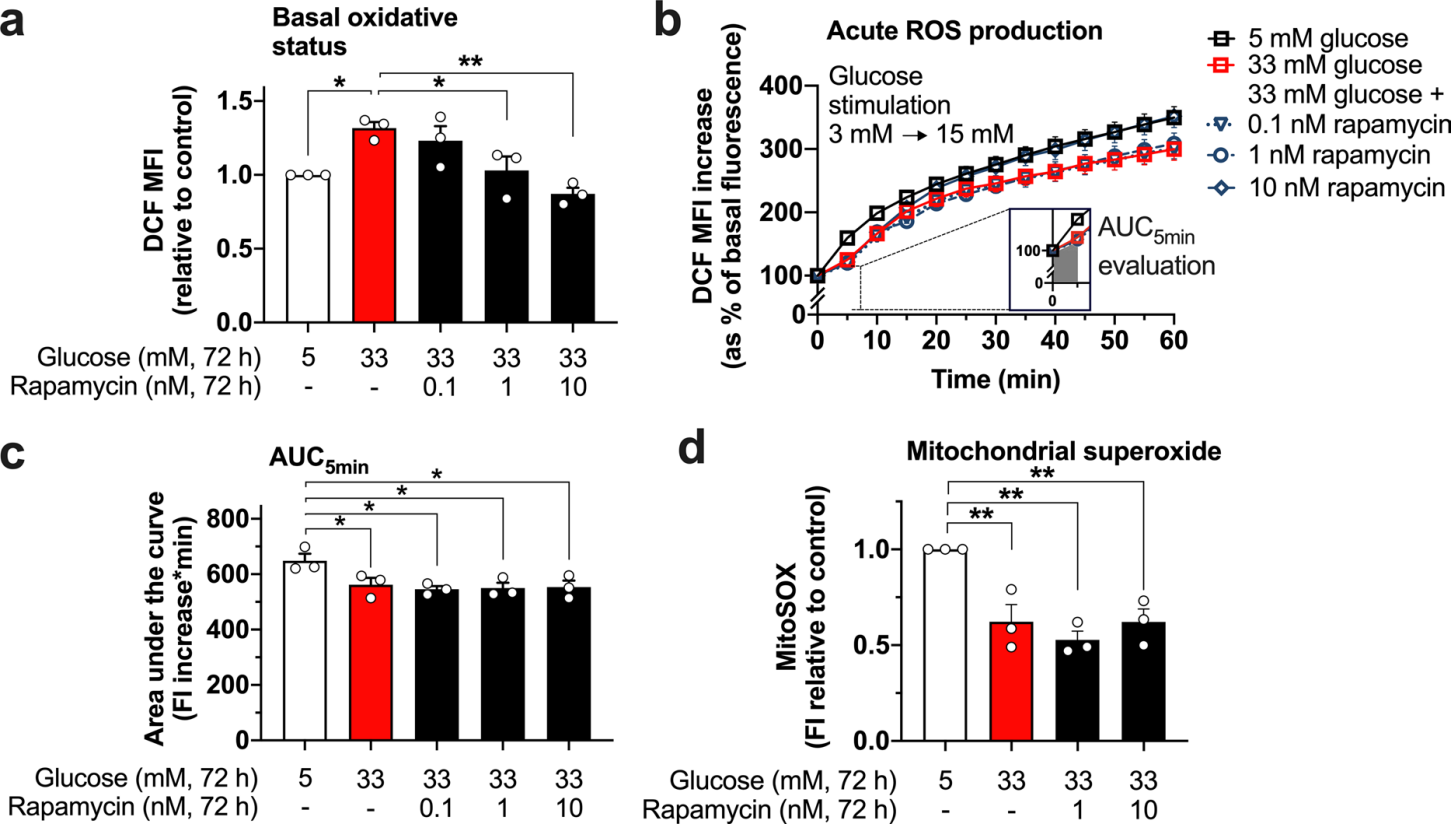
Effect of glucotoxicity and mTOR inhibition on the oxidized status, and mitochondrial superoxide accumulation of MIN6 cells. After a 72 h-exposure of the cells to medium containing 5 or 33 mM glucose and 0.1, 1 or 10 nM rapamycin, DCF fluorescence was measured. Rapamycin dose-dependently reduces the basal oxidized status of MIN6 cells under glucotoxic conditions (33 mM glucose) (**a**). The relative-to-basal ROS production was assessed by an acute glucose stimulation (15 mM glucose, added immediately after determining basal fluorescence, at *t* = 0 min) and monitored every 5 min over a total period of 60 min (**b**). The area under the curve (AUC) of the accumulated ROS during the first 5 min of the glucose stimulation was calculated, and shows that the decreased acute reactivity of cells exposed to glucotoxicity is not prevented by rapamycin (**c**). Mitochondrial superoxide levels after 72 h-culture in glucotoxic conditions are decreased compared to control regardless of the presence of rapamycin (**d**). MFI: mean fluorescence intensity. The circles indicate the number of independent experiments performed for each condition. *p < 0.05; **p < 0.01

Despite ROS signaling being proven essential for the function of pancreatic beta-cells, it is widely accepted that an excess of ROS is deleterious and leads to cell damage and dysfunction. Therefore, we investigated whether ROS play a role in the induction of beta-cell senescence and how mTOR inhibition with rapamycin influences this parameter. The oxidative state of MIN6 cells, detected by DCF fluorescence, is significantly higher after the exposure to 33 mM glucose for 72 h compared to that of cells cultured in medium with 5 mM glucose. Inhibition of mTOR prevents this effect in a dose-dependent manner (Fig. 3a). Next, we assessed how the DCF fluorescence is influenced acutely by switching from a non-stimulatory (3 mM) to a stimulatory (15 mM) glucose concentration in cells previously exposed to glucotoxicity in the absence or presence of rapamycin. Remarkably, the fast reactivity of the cells to an acute glucose stimulus is blunted and the oxidant status is altered in cells stressed by glucotoxicity (Fig. 3b). The significantly reduced DCF fluorescence in response to the acute elevation of glucose (evaluated as area under the curve, AUC, for the first 5 min after elevating the glucose concentration) in MIN6 cells pre-treated with 33 mM glucose *vs*. control cells may indicate a decrease in the acute cellular ROS generation after glucose stimulation (Fig. 3c). This alteration on the responsiveness to glucose is not prevented by 0.1 and 1 nM rapamycin. Of note, the late phase in the kinetics of the DCF fluorescence (min 20 to 60) seems to be normalized by the high concentration of 10 nM rapamycin.

Mitochondrial activity plays a key role for insulin secretion. To complement our data, MitoSox was used as an indicator for mitochondrial superoxide accumulation. After 72 h-culture in glucotoxic medium, MitoSox fluorescence levels of MIN6 cells are significantly decreased, most likely indicating impaired mitochondrial function with a reduction in superoxide production as a by-product of mitochondrial respiration. In contrast to the protective effect of rapamycin against glucotoxicity-mediated changes in the general cellular oxidant status, the inhibition of mTOR does not counteract the influence of glucotoxicity on mitochondrial ROS generation (Fig. 3d).

### Glucotoxicity disturbs cytosolic calcium homeostasis and electrical activity in pancreatic islets in an mTOR-independent manner

**Fig. 4:**
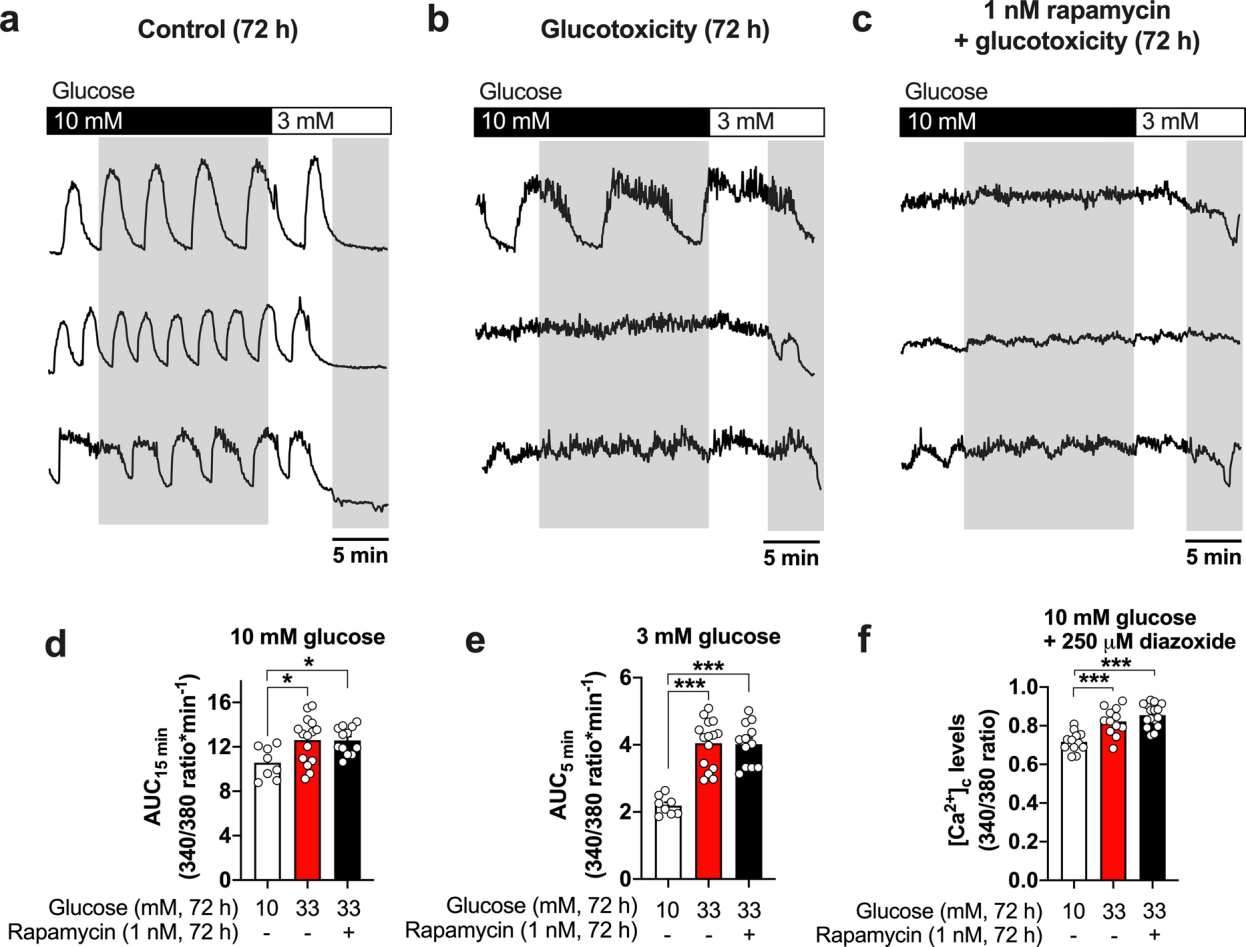
Glucotoxicity disturbs intracellular calcium homeostasis in pancreatic islets, and mTOR inhibition does not prevent this effect. Representative recordings of calcium oscillations of islets cultured for 72 h under control (**a**), glucotoxic (**b**), or glucotoxic conditions in the presence of 1 nM rapamycin (**c**) are shown. Stimulation of the islets with a bath solution containing 10 mM glucose evokes the classical calcium oscillations in controls (**a**), which is clearly disturbed after the exposure to 33 mM glucose (**b**). Rapamycin does not prevent this effect (**c**). Evaluation of the area under the curve (AUC, shadowed parts in exemplary traces) indicates an increase in the cytosolic calcium at 10 mM glucose (**d**) or 3 mM glucose (**e**) after 72 h of glucotoxic culture with or without rapamycin. Acute application of diazoxide (250 µM) does not reduce the calcium concentration to the control level in islets pre-treated with 33 mM glucose in the presence or absence of rapamycin (**f**). Circles indicate the number of islets evaluated from at least three independent mouse preparations (~ 9-months of age, female mice). *p < 0.05; ***p < 0.001

Our data indicate beneficial effects of rapamycin against the induction of the senescence markers p16^INK4a^ and SA-beta-gal in beta-cells but only a partial protection regarding the alterations in oxidative status provoked by the exposure to glucotoxicity. To get more insight into the functional consequences of mTOR inhibition, the cytosolic calcium concentrations and its oscillatory patterns as well as electrical activity were evaluated. Control islets cultured in standard medium show the characteristic oscillatory patterns of calcium when perifused with a bath solution containing a stimulatory glucose concentration of 10 mM. As expected, these calcium oscillations completely cease when the glucose concentration is reduced to 3 mM. The exposure to glucotoxicity causes an evident disturbance of the calcium oscillations, i.e., mostly a lack of oscillations or abnormally prolonged oscillations at 10 mM glucose, indicative of hyperactive beta-cells. Moreover, in contrast to the control islets, the acute lowering of the glucose concentration from 10 to 3 mM does not result in a sharp decrease in cytosolic calcium to the basal level but calcium remains at a plateau followed by a slow and less pronounced decline. Supplementation of the glucotoxic culture medium with 1 nM rapamycin is not able to prevent these glucotoxicity-mediated alterations in the calcium oscillations (Fig. 4a–c, three representative traces are shown for each condition). Data were evaluated as AUC of the traces under stimulatory glucose (10 mM, last 15 min before switching bath solution to a glucose concentration of 3 mM, Fig. 4d) or non-stimulatory (3 mM, last 5 min of each experiment, Fig. 4e) glucose concentration.

In order to elucidate whether the differences in the cytosolic calcium induced by glucotoxicity include alterations in intracellular calcium storage and/or transport, calcium levels were analyzed in the presence of diazoxide (250 µM, 10 mM glucose bath solution, Fig. 4f). Our data show that the cytosolic calcium of glucotoxicity-stressed islets remains elevated during the acute application of diazoxide suggesting an impairment independent of the function of L-type calcium channels and membrane depolarization. Rapamycin treatment also fails to prevent this effect of glucotoxicity.

**Fig. 5:**
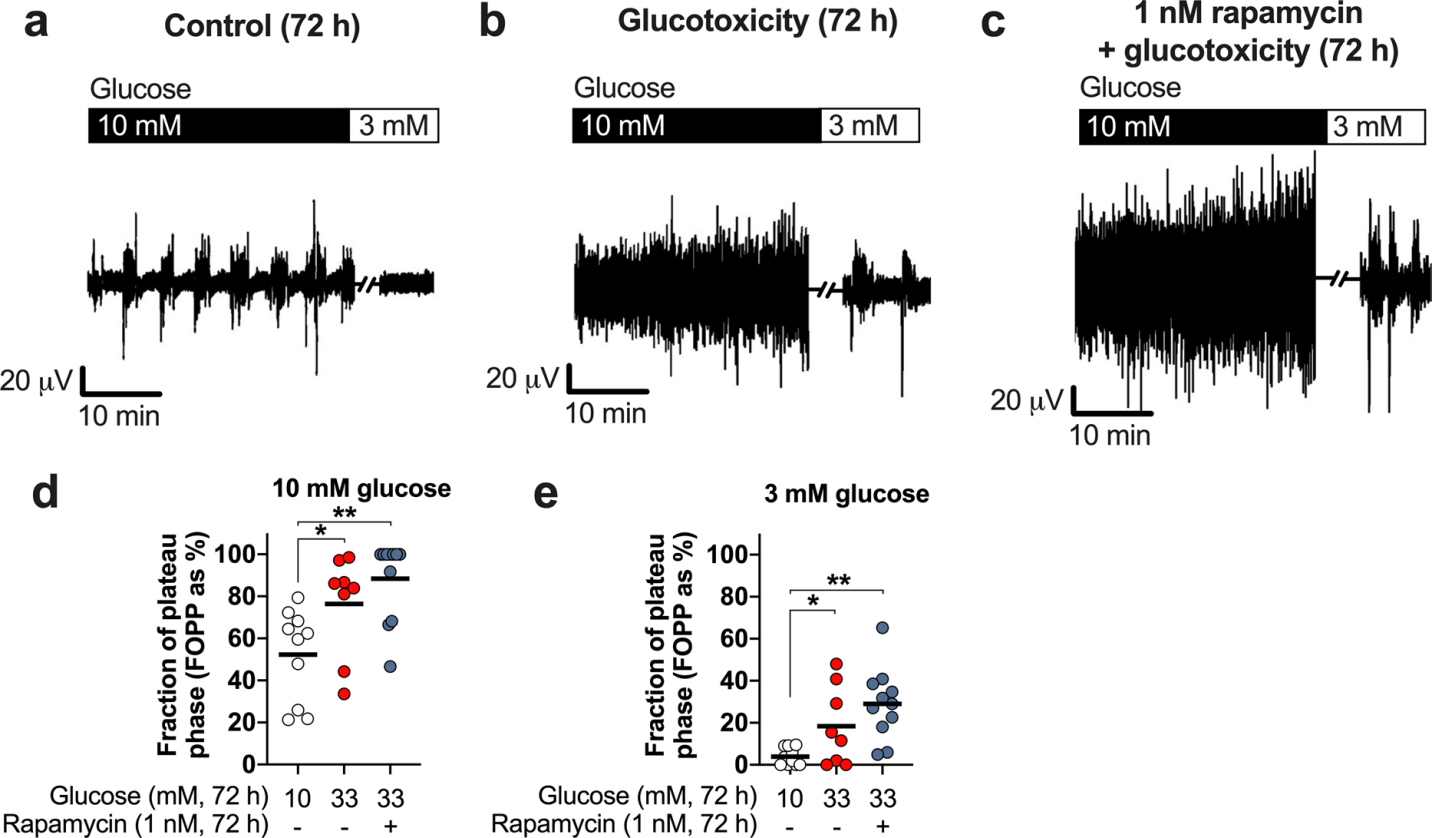
Lack of effect of rapamycin on the electrical activity of islets exposed to glucotoxicity. The determination of electrical activity of pancreatic islets indicates that, after a 72 h-exposure to glucotoxicity, islets become electrically hyperactive (**b**) compared to islets cultured under standard conditions (**a**) as indicated by the evaluation of the time with electrical activity (FOPP: fraction of plateau phase; **d**−**e**). Inhibition of mTOR with rapamycin (1 nM) does not protect against this glucotoxicity-mediated alteration (**c**−**e**) but even trends to further elevate the FOPP. Circles indicate the number of islets evaluated from four independent mouse preparations (~ 8-months of age, female mice). *p < 0.05; **p < 0.01

As oscillations in intracellular calcium are driven by the electrical activity of the islet, islets cultured on microelectrode arrays (MEAs) were acutely stimulated with 10 mM glucose after 72 h culture in control medium, 33 mM glucose medium, or 33 mM glucose medium in the presence of 1 nM rapamycin. Stimulation with glucose evokes the typical pattern of electrical burst activity interspaced with electrically silent periods in control islets. As expected, the electrical activity is silenced by switching to a bath solution containing 3 mM glucose (Fig. 5a). The time period with spike activity (fraction of plateau phase, FOPP) was used for evaluation. In line with the data obtained for intracellular calcium, islets exposed to glucotoxicity become hyperactive under stimulatory or non-stimulatory glucose concentrations (Fig. 5b, d and e) and rapamycin does not prevent this (Fig. 5c, d and e). A subtle, non-significant increase in electrical activity is observed in islets treated with rapamycin compared to glucotoxicity (compare blue *vs.* red dots in Fig. 5d and e).

### Inhibition of mTOR attenuates insulin hypersecretion promoted by glucotoxicity involving a modulation of exocytosis-related genes

**Fig. 6:**
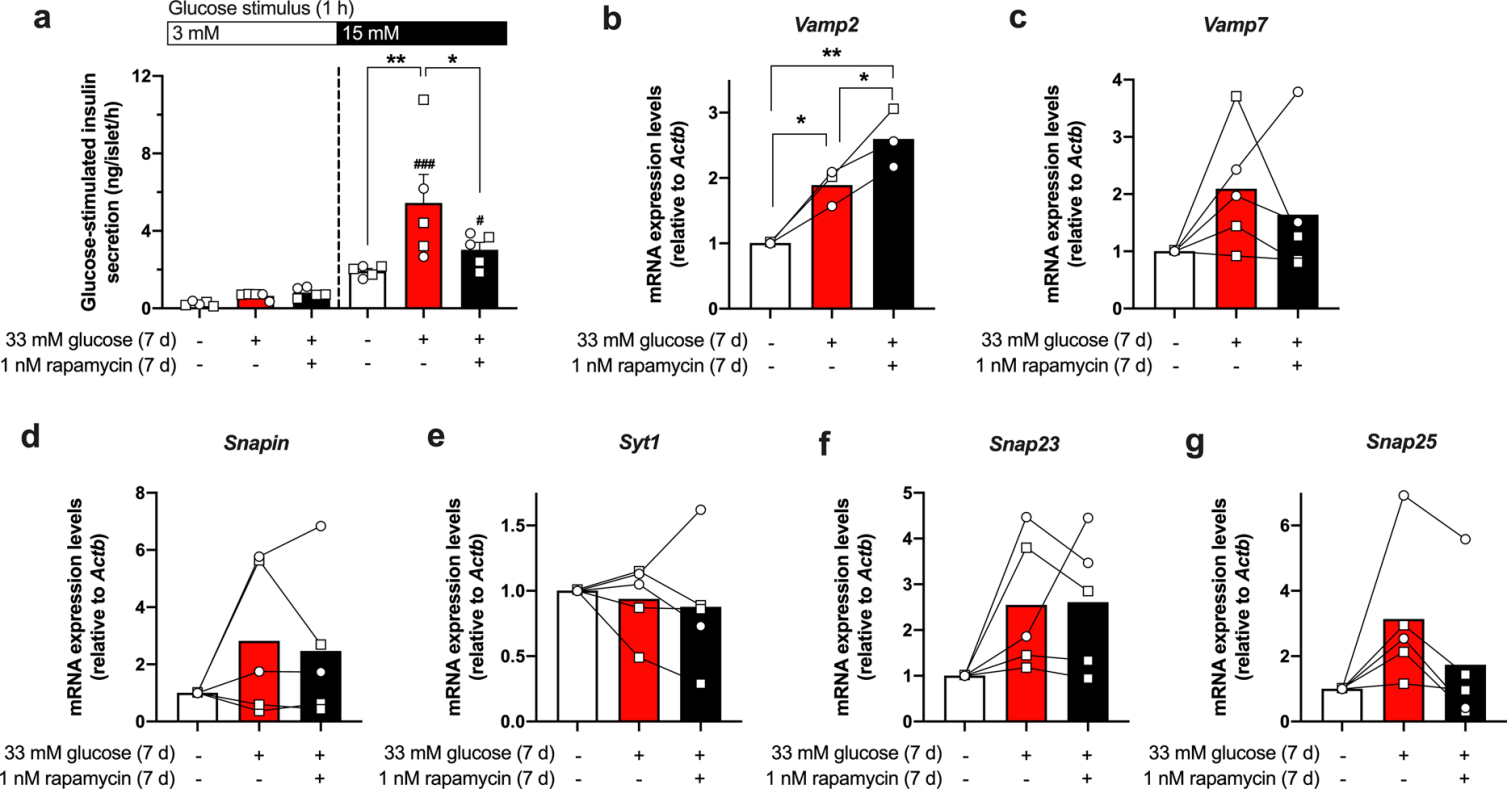
Influence of mTOR inhibition on the glucose-stimulated insulin secretion and the expression of exocytosis-related genes in mouse pancreatic islets exposed to glucotoxicity. After a 7-days exposure to 33 mM glucose, mouse islets develop an insulin hypersecreting phenotype in response to acute stimulation with 15 mM glucose compared to control islets. Rapamycin partly prevents this effect without influencing the basal secretion at 3 mM glucose (**a**). The expression of exocytosis-related genes is heterogeneously upregulated by glucotoxicity (**b**−**g**) and co-culture with rapamycin further increases *Vamp2* levels (**b**) but tends to normalize *Vamp7* (**c**) and *Snap25* (**g**). Circles and squares indicate the number of independent female or male mouse preparations, respectively (~ 3-months of age for GSIS; and ~ 8-months of age for RT-qPCR assays). **a** − **g**: *p < 0.05; **p < 0.01; **a**: ^#^*p* < 0.05, ^###^*p* < 0.01 *vs*. control with 3 mM glucose

To evaluate whether inhibition of mTOR exerts any effects on the secretory capacity of islets, glucose-stimulated insulin secretion (GSIS) and insulin content were determined in a static incubation (1 h) of the islets under stimulatory (15 mM) or non-stimulatory (3 mM) glucose concentration. In agreement with the data for electrical activity and intracellular calcium, GSIS at 15 mM glucose is significantly higher after a 7-days glucotoxic culture period compared to control islets, whereas secretion in response to 3 mM glucose only tends to rise. Noteworthy, despite the lack of protection on the islet´s electrophysiological features and calcium homeostasis, the inhibition of mTOR by rapamycin (1 nM) significantly attenuates the hypersecretory phenotype (Fig. 6a, black *vs*. red bar). Regarding the insulin content, the rapamycin treatment has no significant effects (glucotoxicity: 58.16 ± 19.65 ng/islet, glucotoxicity + rapamycin: 44.7 ± 5.28 ng/islet, n = 5).

The rapamycin-induced reduction of insulin release could be due to changes in the exocytotic machinery. To analyze the effects of rapamycin on exocytosis-related genes, the mRNA transcription levels of *Vamp2*, *Vamp7*, *Snapin*, *Syt1*, *Snap23* and *Snap25* in islets exposed to the glucotoxic condition in presence of 1 nM rapamycin for 7 days *vs*. control was determined (Fig. 6b−f; Online Resource Figure S3). The average expression level of *Vamp2* is significantly elevated by culture under glucotoxicity and further upregulated by co-culture with rapamycin (Fig. 6b). The mRNA for the other proteins, except *Syt1*, also tend to rise after glucotoxic culture (Fig. 6c−g). Rapamycin counteracts this trend for *Vamp7* (Fig. 6c, 3 out of 5 experiments), *Snap23* (Fig. 6f, 4 out of 5 experiments) and *Snap25* (Fig. 6g, all experiments), whereas the influence on *Snapin* is highly variable.

## Discussion

Senescence has gained notorious scientific interest due to its involvement in the aging progression and development of age-related diseases, such as T2D. Notwithstanding, the importance of senescence for insulin-producing beta-cells remains yet to be clearly defined. Our study aims to correlate and to qualitatively characterize mechanisms of islet cell adaptation (or failure) during cell stress and induction of known senescence markers, as well as the significance of mTOR pathway for these functional and molecular alterations. This basic investigation is an essential prerequisite for addressing current knowledge gaps in biogerontology concerning the efficacy of any interventional strategy interacting with repair and recovery during the aging process (Rattan 2024). Likewise, identifying whether the generic biomarkers usually associated with senescence are applicable for the pancreatic beta-cells, which are known to possess a low replication rate, requires thorough assessment. We focused on investigating senescence induction in primary mouse beta-cells by employing two of the most widely studied markers for senescence, SA-beta-gal and p16^INK4a^. To enhance our understanding of how alterations in islet function may be related to senescence- and aging-associated conditions, we use an experimental scenario that induce metabolic and oxidative stress without severely damaging the islet cells. Regarding the experiments where cells are exposed to glucotoxicity, the islets adapt to this challenge by hyperexcitability and hypersecretion. In the context of T2D, this is usually accepted as a relatively early disease state at which compensatory processes are activated. In the context of aging accelerated senescence may occur due to over-activation of signaling pathways such as mTOR. Certainly, this model—as all in vitro models with isolated tissue or organs—does not entirely reflect the complex dynamics of senescence-associated metabolic interactions (or failure) in the whole organism (Rattan 2024). As the induction of SA-beta-gal and the hypersecretory phenotype in response to glucotoxicity occurred in islets of male and female mice, the data of both sexes were pooled. Certainly, this does not exclude that pancreatic islets per se or their adaptation to metabolic demands during aging show sex-dependent differences. In an ongoing study, we compare the potential sex-related behavior and the influence of mTOR in islets of different ages without further challenges to close this gap of knowledge.

The SA-beta-gal is a lysosomal protein regularly used to identify senescent cells (Lee et al. 2006). In a recent study, the histological analysis of pancreata from adult middle-aged mice (9-months-old) revealed that the endocrine pancreas displays positivity for SA-beta-gal staining, possibly due to the high proportion of cells in a post-mitotic state (Raffaele et al. 2020). In our experiments, similar to those findings, mouse pancreatic islet-cells exhibit a basal level of C_12_FDG fluorescence indicating beta-gal activity. Glucotoxicity has been suggested to exert deleterious effects on beta-cells by the promotion of oxidative stress (Huang et al. 2017; Tang et al. 2007). However, our data indicate that hydrogen peroxide-induced oxidation is not enough for triggering a senescent phenotype as indicated by SA-beta-gal. Promoting mTOR activation by insulin also does not lead to a rise in SA-beta-gal activity. This contrasts to the insulin-mediated and mTOR-dependent induction of senescence markers reported for hepatocytes (Baboota et al. 2022), adipocytes (Li et al. 2021) and neuronal cells (Chow et al. 2019).

The anabolic protein kinase mTOR serves as a central hub for the integration of nutrient and growth factor signals, regulating mainly cell metabolism, proliferation, and survival (Ardestani et al. 2018; Liu and Sabatini 2020). Moreover, this pathway has been proven essential for beta-cell biology since the knockout of a negative mTOR regulator, the tuberous sclerosis complex 1 (TSC1), promotes a higher beta-cell mass and improves glucose tolerance in rodents (Blandino-Rosano et al. 2012; Ding et al. 2017). Notably, in our experiments, mTOR inhibition with rapamycin in the low nanomolar range prevents the activation of SA-beta-gal by glucotoxicity in adult mouse islet-cells. In line with our observations, the same, low rapamycin concentration is enough to protect endothelial cells against the induction of some senescence biomarkers, including SA-beta-gal (Khor and Wong 2018). Other studies reporting an effect of rapamycin for different cell types (Bai et al. 2021; Nie et al. 2021; Sasaki et al. 2020), usually applied higher concentrations of the drug, where the likelihood for adverse effects rises (Barlow et al. 2013; Deblon et al. 2012).

The p16^INK4a^ protein acts as a tumor suppressor molecule and is involved in the regulation of the cell cycle by inhibiting cyclin-dependent kinases (Safwan-Zaiter et al. 2022). The level of p16^INK4a^ protein was shown to increase with age in mouse pancreatic islets (Chen et al. 2009; Tschen et al. 2009). Expression was also immunodetected in islets from healthy young animals as well as in islets from non-diabetic human donors (Thompson et al. 2019; Tschen et al. 2009). This suggests that the presence of the protein might be related to the differentiated stage and to functional maturity of the beta-cells (Helman et al. 2016). In our study, p16^INK4a^ immunostaining is not clearly altered by a 72-h exposure of islet cells to high-glucose medium but increases significantly after 7 days of culture under this condition. In accordance with our results, Lee and colleagues observed an increase in p16^INK4a^ in the rat insulinoma-derived INS-1 cell line after exposure to glucotoxicity. Contrasting to our data showing a predominantly nuclear localization of p16 ^INK4a^ in primary islet cells, the protein was found mostly in the cytosol in the INS-1 cells (Lee et al. 2020). The p16^INK4a^ localization in malignant cells is suggested to be correlated to the overall survival and progression-free interval in patients with head and neck cancer, with a cytosolic p16^INK4a^ localization being associated to a less favorable prognosis (Zhao et al. 2012). To the best of our knowledge, the importance of p16^INK4a^ localization has not been studied thoroughly, and experimental data for regulation of distribution and translocation of p16^INK4a^ in beta-cell compartments is non-existent. The turnover of p16^INK4a^ protein is modulated by autophagy (Coryell et al. 2020). Our observation of a significant reduction of the protein content in the cytosolic and the nuclear compartment of islet cells treated with rapamycin might indicate an activation of autophagy-related lysosomal degradation of p16^INK4a^. Of note, rapamycin decreases p16^INK4a^ immunofluorescence already in the presence of 10 mM glucose, which is the standard culture condition for murine islets. This provisionally suggests that mTOR inhibition has the potential to interact with p16^INK4a^-mediated senescence mechanisms independent of a disease state, but further studies are necessary to address this issue in detail.

An elegant study performed by Li and colleagues (2014) indicated that calcium homeostasis deteriorates with the aging of beta-cells, a defect that seems to rely on a progressive decline of mitochondrial function (Barker et al. 2015; Li et al. 2014). Likewise, beta-cell calcium signaling was reported to be altered under pathological conditions such as insulin resistance and T2D (Gilon et al. 2014; Gonzalez et al. 2013). A proper calcium handling is highly relevant for electrically excitable cells, since the electrical activity is closely coupled to changes in the cytosolic calcium concentrations (Drews et al. 2010). In our glucotoxic model, the islets are functionally in a compensatory stage, i.e., acute stimulation with glucose results in elevated electrical activity, the typical bursts of depolarization are changing to a pattern of constant action potential firing leading to a rise in the overall intracellular calcium and an insulin hypersecreting phenotype.

Remarkably, hypersecretion is partially prevented by addition of rapamycin to the glucotoxic culture medium. In agreement with our finding, a 16-week intervention with a weekly injection of rapamycin in mice previously fed a high-fat diet for 20 weeks, reduced the serum insulin levels and promoted significant weight loss (Chang et al. 2009). These data underline the potential of mTOR inhibitors to reverse an insulin hypersecretory phenotype. The reversal of insulin hypersecretion in our in vitro model is not mediated by a reversion of glucotoxicity-induced changes in the glucose-sensitivity of the classical stimulus-secretion cascade. An intriguing aspect that remains to be clarified by long-term experiments with “aged islets” at different timepoints is, whether interfering with the hypersecreting phenotype by modulating mTOR proves to be beneficial, neutral or detrimental for the dynamic regulation of blood glucose during the aging process with its heterogenous demands (Rattan 2024).

Besides functional evaluations, we assessed the expression of several vesicular and plasma membrane-targeted exocytosis-related genes to understand how rapamycin is able to normalize the GSIS. We observed that mRNA expression levels of *Vamp2*, *Vamp7*, *Snapin*, *Snap23*, and *Snap25* exhibit a 2-to-threefold average increase after the exposure to glucotoxicity. In islets isolated from rodent models of diabetes characterized by reduced secretion and content of insulin, VAMP2 and SNAP25 protein is decreased (Nagamatsu et al. 1999; Zhang et al. 2002), which is supportive for the hypothesis that upregulation of the expression of these proteins contributes to the elevated exocytosis we observed after glucotoxic culture. The exposure of rat islets to glucotoxicity for 7 days resulted in a dramatic downregulation of the VAMP2 protein, a tendency to reduced levels of SNAP25, but a marked increase in SNAP23 levels confirming the critical role of these proteins during beta-cell impairment (Torrejon-Escribano et al. 2011). As GSIS of the rat islets does not show hypersecretion *vs.* control, but is already decreasing in the study of Torrejon-Escribano et al*.,* the discrepancies to our results most likely indicate different stages of islet exhaustion. Another reason might be post-transcriptional regulatory mechanisms, e.g., increased protein turnover inducing a rise in gene transcription. Other targets like *Vamp7*, *Snapin* and *Syt1* have been less studied and their sensitivity to glucotoxicity is not known. Recent reports indicate a role for VAMP7 in autophagic processes and an increased level has been found in islets of high-fat diet-fed and *db*/*db* mice. Interestingly, the authors observed a significant reduction in the amount of insulin secreted from beta-cell specific VAMP7 knockout islets (Aoyagi et al. 2018). In our experiments, the mTOR inhibition with rapamycin led to a normalization of *Snap25* and a tendency to decreased expression of *Vamp7* but increased the levels of *Vamp2*. Related with this, in a recent study by Kang and colleagues (2023), neurobehavioral deficits were analyzed in aged rats subjected to anesthesia plus surgery and treated with rapamycin. Rapamycin restored the SNAP25 and VAMP2 protein levels in the hippocampus of treated animals (Kang et al. 2023). Our data suggest similar rapamycin effects regarding *Snap25* expression. The mRNA levels of *Snapin* and *Syt1*, which both interact with SNAP25 (Nakajima-Nagata et al. 2004; Song et al. 2011; Zhuang et al. 2023), remain stable after the exposure to glucotoxicity and rapamycin treatment has no influence on these genes. This implies that the regulation of specific exocytosis-related targets is enough to extenuate the insulin hypersecretory phenotype developed by the exposure to glucotoxicity.

In conclusion, our data indicate that senescence of primary mouse islet cells is promoted by the chronic exposure to glucotoxicity resulting in hypersecretion and alterations in the transcription of exocytosis-regulating genes. Inhibition of mTOR prevents the induction of the senescence markers SA-beta-gal and p16^INK4a^ and attenuates the glucotoxicity-mediated change in insulin secretion by modulating the pattern of genes relevant for exocytosis. These changes occur independent of alterations in the electrical features and calcium homeostasis promoted by the glucotoxic conditions.

## Limitations of the study

Although the mTOR-targeted approach changes senescence-associated parameters, extrapolation of the results to the complex situation in the aging organism is impossible. Whether influencing mTOR-dependent signaling not only interacts with disease but also promotes a healthier survival and adaptive capacity of pancreatic islets during aging is the next step to be investigated in long-term studies.

Our experiments were performed in primary mouse islet cells which comprise mostly beta-cells (~ 80%), but whether the subtypes of pancreatic endocrine cells exhibit different susceptibility to senescence induction/resistance, or if these effects are species-dependent, needs to be explored in the future.

We studied the influence of mTOR signaling solely for rapamycin that predominantly but not exclusively targets mTORC1. Elucidating the involvement of mTORC1 or mTORC2 complexes during senescence promotion in primary beta-cells in more detail requires additional methodological approaches.

Finally, the senescence-associated secretory phenotype (SASP) has been shown to be regulated through mTOR pathways in some cell types. If rapamycin can prevent the development of SASP in aging or diabetic islet cells should be addressed in future studies.

## Supplementary Information

Below is the link to the electronic supplementary material.

Supplementary file1 (PDF 1286 KB)

## Data Availability

The data that support the findings of this study are available from the corresponding author upon reasonable request.
