# Supplementary material for: Inhibition of mTOR prevents glucotoxicity-mediated increase of SA-beta-gal, p16INK4a, and insulin hypersecretion, without restoring electrical features of mouse pancreatic islets
Source: Biogerontology. 2024 May 15;25(5):819–36. doi: 10.1007/s10522-024-10107-9 (PMC11374829; doi:10.1007/s10522-024-10107-9)
Supplement: Supplementary file 1 — Supplementary file1 (PDF 1286 KB) [file 10522_2024_10107_MOESM1_ESM.pdf]

Supplementary material:

Inhibition of mTOR prevents glucotoxicity-mediated increase of SA-beta-gal, p16^{INK4a}, and insulin hypersecretion, without restoring electrical features of mouse pancreatic islets

Tereso J. Guzmán[1,2,*], Nina Klöpper[1], Carmen M. Gurrola-Díaz[2], Martina Düfer[1,*]

[1] University of Münster, Institute of Pharmaceutical and Medicinal Chemistry, Department of Pharmacology, Corrensstraße 48, 48149 Münster, Germany.

[2] Universidad de Guadalajara, Instituto de Investigación en Enfermedades Crónico-Degenerativas, Departamento de Biología Molecular y Genómica, Centro Universitario de Ciencias de la Salud, 44340 Guadalajara, Jalisco, México.

***Corresponding authors:**

Prof. Dr. Martina Düfer

Address: Corrensstraße 48, 48149 Münster, Germany. Tel.: +49 251 83 33339, Fax: +49 251 83 32144. E-mail: martina.duefer@uni-muenster.de,

ORCID: https://orcid.org/0000-0003-1572-9235

Dr. Tereso J. Guzmán

Address: Corrensstraße 48, 48149 Münster, Germany. Tel.: +49 251 83 33371.

E-mail: goozman57@hotmail.com, ORCID: https://orcid.org/0000-0002-1900-5931

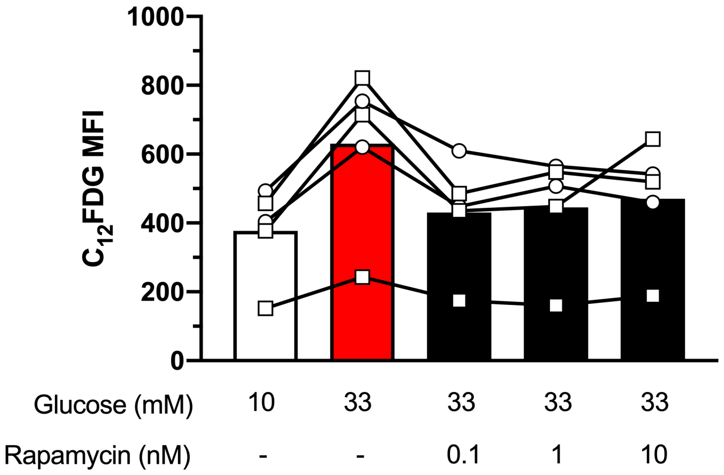

Fig S1. Raw C_{12}FDG fluorescence values in pancreatic islet cells exposed to glucotoxicity in the presence of rapamycin or vehicle. After 72 h of incubation under the indicated conditions, the level of SA-beta-gal activity was assessed with the fluorogenic substrate C_{12}FDG. The number of independent female and male mouse preparations is indicated by the circles and squares, respectively. The lines correlate the outcome in every condition by experiment. MFI: mean fluorescence intensity.

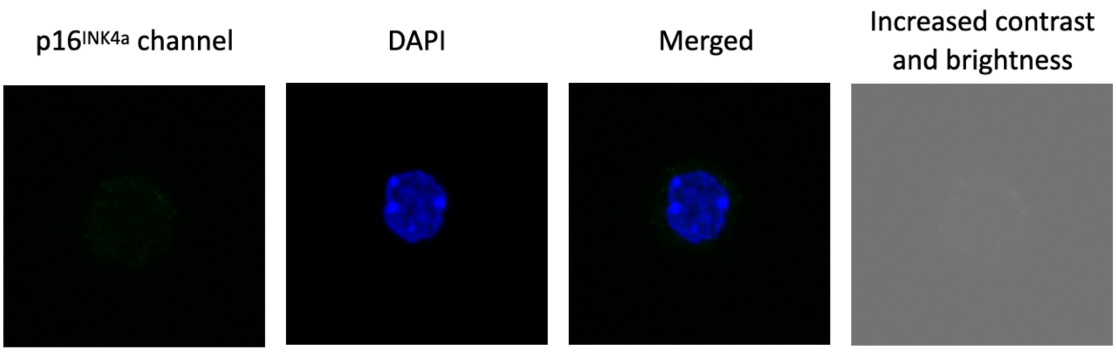

Fig S2. p16^{INK4a} immunostaining negative control in mouse pancreatic islet cells. The cells were stained with the standardized immunostaining protocol for p16^{INK4a}, replacing the primary antibody with blocking solution (10% goat serum). For counterstaining, the nuclei were revealed with DAPI and images were acquired with a confocal microscope.

Table S1. Primers employed for gene expression analyses by RT-qPCR.

Gene	Primer	5'-3' nucleotide sequence
Vamp2	Forward	GTTTGCTTCCCTTACCCCGT
	Reverse	CAGTTGAGTGCCCCACATGA
Snapin	Forward	TCTCCAAGCAAATAAGCACGG
	Reverse	AACAAAACCCAAAAGGTAGAGGC
Snap23	Forward	GTCCGAGAACTGTGGAGGCT
	Reverse	GGATACTCTGTCCCGCTGACTA
Snap25	Forward	TGTGCTGTCTTTGGTTCCTCA
	Reverse	GCAGGTTTTGCTGGTATGACT
Syt1	Forward	GCCCGACAAAAAGAAGAAGTT
	Reverse	CCGAGTATGGCACCTTGAAA
Vamp7	Forward	ACCTTCGCCCCTCAGTCAATA
	Reverse	CCCTGGCAACAACAGCAAAAAG
Actb	Forward	TCGTACTCCTGCTTGCTGAT
	Reverse	AGATTACTGCTCTGGCTCCTA

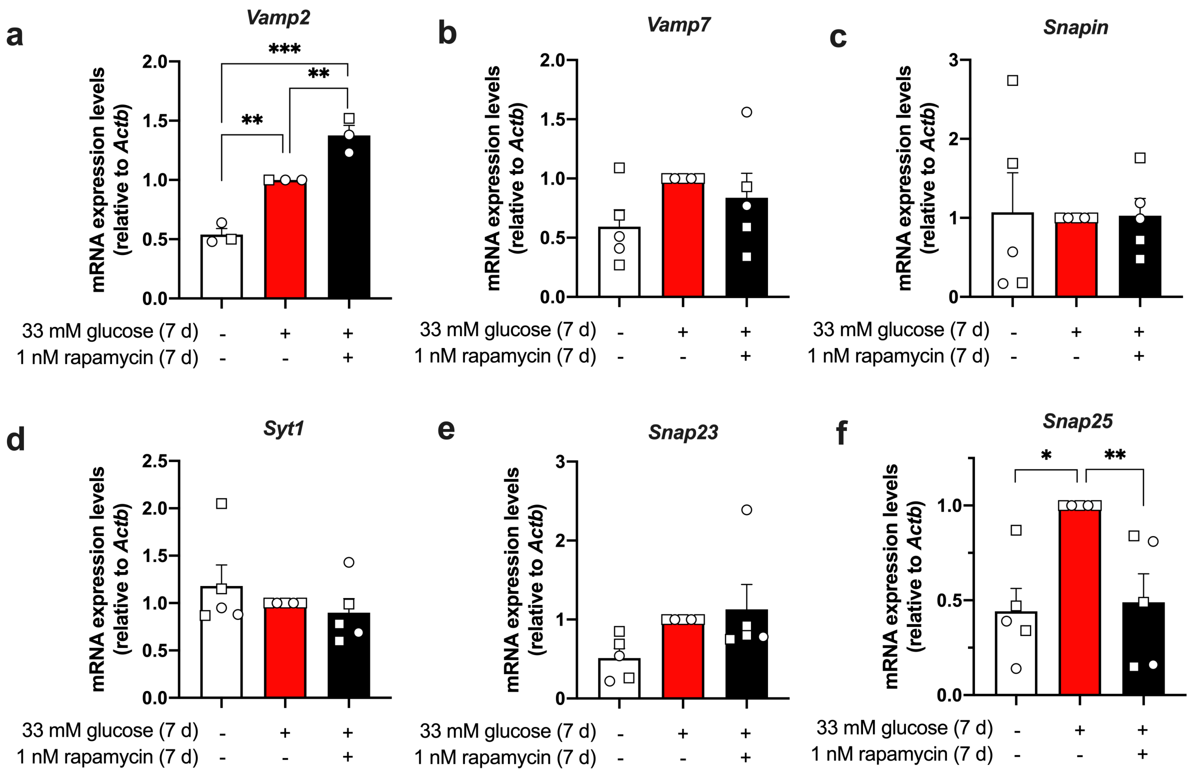

Fig S3. Gene expression analysis of exocytosis-related genes in mouse pancreatic islet cells exposed to glucotoxicity in the presence or absence of rapamycin for 7 d. We compared the gene expression of *Vamp2* (**a**), *Vamp7* (**b**), *Snapin* (**c**), *Syt1* (**d**), *Snap23* (**e**) and *Snap25* (**f**) using the gene expression under glucotoxic conditions (red bars) as reference mRNA expression levels. The treatment with 1 nM rapamycin (black bars) in the presence of glucotoxicity, modulated the gene expression of *Vamp2* and *Snap25*, promoting a significant upregulation and downregulation, respectively. *Actb* was used as a housekeeping gene for the expression analyses. The number of independent male and female mouse preparations is indicated by the squares and circles, respectively. *$p < 0.05$; **$p < 0.01$; ***$p < 0.001$.
